# Signaling Pathways Regulating Thermogenesis

**DOI:** 10.3389/fendo.2021.595020

**Published:** 2021-03-26

**Authors:** Chihiro Tabuchi, Hei Sook Sul

**Affiliations:** Department of Nutritional Sciences and Toxicology, University of California, Berkeley, Berkeley, CA, United States

**Keywords:** thermogenesis, brown adipose tissue, browning/beiging, b3-adrenergic signaling, UCP1, insulin/IGF1 signaling, thyroid hormone, TGFβ superfamily

## Abstract

Obesity, an excess accumulation of white adipose tissue (WAT), has become a global epidemic and is associated with complex diseases, such as type 2 diabetes and cardiovascular diseases. Presently, there are no safe and effective therapeutic agents to treat obesity. In contrast to white adipocytes that store energy as triglycerides in unilocular lipid droplet, brown and brown-like or beige adipocytes utilize fatty acids (FAs) and glucose at a high rate mainly by uncoupling protein 1 (UCP1) action to uncouple mitochondrial proton gradient from ATP synthesis, dissipating energy as heat. Recent studies on the presence of brown or brown-like adipocytes in adult humans have revealed their potential as therapeutic targets in combating obesity. Classically, the main signaling pathway known to activate thermogenesis in adipocytes is β_3_-adrenergic signaling, which is activated by norepinephrine in response to cold, leading to activation of the thermogenic program and browning. In addition to the β_3_-adrenergic signaling, numerous other hormones and secreted factors have been reported to affect thermogenesis. In this review, we discuss several major pathways, β_3_-adrenergic, insulin/IGF1, thyroid hormone and TGFβ family, which regulate thermogenesis and browning of WAT.

## Introduction

Brown adipose tissue (BAT) is specialized in heat production through non-shivering thermogenesis by burning fuels such as fatty acids (FA) and glucose (1). BAT is crucial for maintaining body temperature especially for infants who have limited ability to perform shivering thermogenesis, due to underdeveloped skeletal muscle (2). In adults, BAT contributes to energy expenditure as indicated by an inverse correlation between the presence of active BAT and central obesity (3, 4). In addition to numerous mitochondria, BAT possesses multilocular lipid droplets that provide FAs for β-oxidation, which drives tricarboxylic acid (TCA) cycle and electron transport chain (ETC) to create proton gradient (5). In contrast, white adipose tissue (WAT) serves as an energy storage and has a unilocular lipid droplet and fewer mitochondria.

Of all tissues, BAT expresses the highest levels of uncoupling protein 1 (UCP1) and UCP1 plays a central role in non-shivering thermogenesis (6). UCP1 acts as a proton transporter in the inner membrane of mitochondria, which allows protons to leak from intermembrane space into the mitochondrial matrix (7). This uncouples the proton gradient from ATP synthesis, dissipating the proton gradient in the form of heat. Therefore, having a large number of mitochondria and multilocular lipid droplets enables BAT to maximize its thermogenic capacity. Although WAT normally has little UCP1 expression, some cells in subcutaneous WAT depots display thermogenic capacity with elevated UCP1 expression upon certain stimulations, the process so-called ‘browning/beiging’ (8). These thermogenic adipocytes are termed ‘beige/brite’ adipocytes, which seem to originate from different precursor cells than brown adipocytes, based on distinct gene signatures (9). However, transdifferentiation of mature white adipocytes to thermogenic beige adipocytes have also been reported (10). It is plausible that these thermogenic adipocytes can be generated by distinct mechanisms.

Classical brown adipocytes originate from a subset of multipotent stem cells in the dermomyotome that express engrailed 1 (EN1), paired box 7 (PAX7) and myogenic factor 5 (MYF5) (11). While the MYF5^+^/PAX7^+^/EN1^+^ progenitor cells can give rise to other cell types, such as skeletal myocytes, they commit to brown adipocyte lineage upon early B cell factor 2 (EBF2) expression (12). Additionally, PR domain containing 16 (PRDM16) stimulates brown adipocyte differentiation over myoblast differentiation through the interaction with peroxisome proliferator activated receptor γ (PPARγ) (13). Ewing Sarcoma (EWS)/Y-box binding protein 1 (YBX1) complex promotes bone morphogenetic protein 7 (BMP7) transcription, which also determines brown adipocyte lineage during development (14). Committed brown precursor cells then differentiate into mature brown adipocytes through a series of transcriptional events (11, 15). ZFP516 stimulates thermogenic gene expression such as UCP1, DIO2 and CIDEA to promote brown adipocyte differentiation. ZFP516 interacts with not only PRDM16, but also with LSD1 in activating thermogenic gene program. ZC3H10 upregulates transcription of genes for mitochondrial biogenesis, such as NRF1 and TFAM (16, 17). EBF2 activates transcription of PPARγ, which promotes expression of numerous lipid metabolic genes, such as fatty acid binding protein (FABP4/aP2), cluster of differentiation 36 (CD36) and lipoprotein lipase (LPL) (18). In addition, EBF2 and PPARγ cooperatively activate transcription of PRDM16. CEBPβ also activates PPARγ transcription to promote adipogenic and thermogenic gene expression (19). PPARγ coactivator α (PGC1α) is another transcriptional co-regulator that interacts with many proteins including PPARγ, PRDM16 and IRF4 to promote BAT development, especially mitochondrial biogenesis through NRF1 and TFAM (15, 20). Some of the aforementioned transcription factors and co-activators such as ZFP516, ZC3H10 and PGC1α also induce thermogenic gene expression upon β_3_-adrenergic stimuli in mature brown and white adipocytes, the latter of which leads to browning.

Environmental cues such as cold exposure, exercise and diet can trigger non-shivering thermogenesis in classical BAT and also browning of WAT, by activating specific signaling cascades within the cell (20, 21). For example, cold temperature stimulates the sympathetic nervous system to release norepinephrine, which then binds β_3_-adrenergic receptor (15). β_3_-adrenergic signaling has a wide range of downstream targets including numerous transcription factors and co-activators that can induce thermogenic genes (22). In addition to the transcriptional regulation, β_3_-adrenergic signaling also increases lipolysis and glucose uptake to support thermogenesis (23). In this review, we discuss underlying signaling pathways that regulate thermogenesis in brown and beige adipocytes, as well as browning of WAT.

## β_3_-Adrenergic Signaling

**Figure 1:**
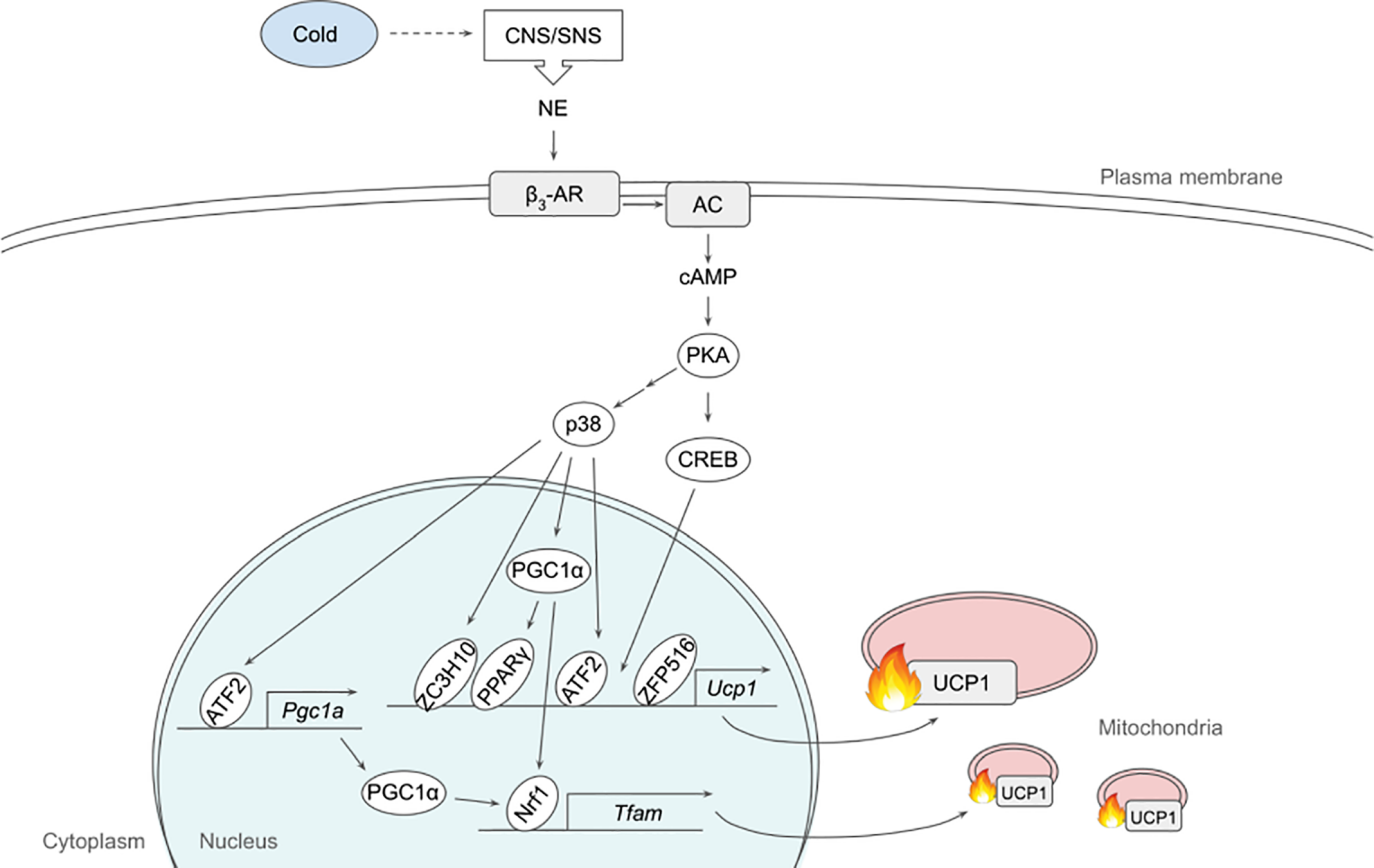
Schematic model of β_3_-adrenergic signaling pathway that promotes thermogenesis in adipocytes. Cold stimulates CNS/SNS to secrete NE that binds β_3_-AR, which then activates AC producing cAMP. cAMP in turn activates PKA that has a variety of downstream targets, including transcription factors to upregulate thermogenic gene expression. See text for details. AC, adenylate cyclase; ATF2, activating transcription factor 2; β_3_-AR, β_3_-adrenergic receptor; CNS, central nervous system; CREB, cAMP response-element binding protein; ETC, electron transport chain; FFA, free fatty acid; IRF4, interferon regulatory factor 4; NE, norepinephrine; NRF1, nuclear respiratory factor 1; PGC1α, peroxisome proliferator activated receptor γ coactivator α; PKA, protein kinase A; PPARγ, peroxisome proliferator activated receptor γ; SNS sympathetic nervous system; TCA, tricarboxylic acid; TFAM, mitochondrial transcription factor A; UCP1, uncoupling protein 1; ZC3H10, zinc finger CCCH-type containing 10; ZFP516, zinc finger protein 516.

The dominant signaling pathway that governs non-shivering thermogenesis in brown and beige adipocytes is β_3_-adrenergic signaling. Upon cold exposure, norepinephrine released from the sympathetic nervous system binds mainly β_3_-adrenergic receptor (β_3_-AR) to induce adrenergic signaling (24, 25) (**Figure 1**). β_3_-AR coupled with Gs activates AC, which in turn produces cAMP for PKA activation. A variety of downstream targets of PKA, such as p38, CREB and HSL, enhances thermogenesis by inducing thermogenic gene expression and/or mobilizing substrate to fuel thermogenesis (26, 27).

p38, a MAP kinase, phosphorylates multiple transcription factors/coregulators, including ATF2 and PGC1α, both of which promote UCP1 transcription (22). In addition, we recently found that ZC3H10, previously known to bind RNA, is phosphorylated by p38 upon cold, activating the thermogenic gene program in adipocytes (17). Specifically, ZC3H10 binds a distal upstream region of the UCP1 promoter for transcriptional activation. ZC3H10 also activates NRF1 and TFAM, which facilitate mitochondrial biogenesis to increase thermogenic capacity of adipocytes. Thus, transgenic mice overexpressing ZC3H10 exhibited increased oxygen consumption, higher BAT temperature and reduced body weight while ZC3H10 knockout mice displayed decreased oxygen consumption, lower BAT temperature and increased body weight. PGC1α, a downstream target of p38, serves as co-activator for PPARγ and NRF1 to induce expression of UCP1 and TFAM, respectively (27, 28). CREB was also shown to bind to the proximal promoter of UCP1 to potentially activate the transcription.

β_3_-adrenergic signaling also increases the expression of ZFP516. ZFP516 recruits LSD1, which demethylases H3K9 at the proximal region of UCP1 promoter to induce UCP1 transcription. Furthermore, ZFP516 directly interacts with PRDM16 at the UCP1 promoter and potentially at other BAT gene promoters. ZFP516 depletion *in vivo* resulted in impaired BAT formation with decreased BAT gene expression and increased muscle gene expression in the presumptive BAT depot, while ZFP516 overexpression increased body temperature, oxygen consumption, browning of WAT, leading to lower body weight. Taken together, ZFP516 is required for BAT development in addition to promoting WAT browning.

Adipose thermogenesis can be enhanced by an increased supply of substrates such as FA and glucose. Upon β_3_-AR activation, PKA phosphorylates HSL, now known as diacylglycerol hydrolase, as well as PLIN on lipid droplets to promote lipolysis. FAs thus produced upon lipolysis can undergo β-oxidation to eventual production of NADH and FADH for ETC, during UCP1-mediated thermogenesis (5, 29). FA also directly binds UCP1 to assist proton influx into the mitochondrial matrix (30). In the fed state, however, FAs as substrates for thermogenesis seem to originate from white adipose tissue lipolysis, although it was also reported that inhibition of intracellular lipolysis suppressed cold-induced non-shivering thermogenesis in BAT, which was compensated by an increased shivering in humans (31, 32). Of note, lipolysis can also be modulated non-adrenergically and this aspect has been extensively reviewed elsewhere (33).

We recently discovered a BAT-specific NADH oxidase, AIFM2, which is required for cold- and diet-induced thermogenesis (34). Upon cold or β_3_-adrenergic stimuli, AIFM2 translocates from lipid droplet to the outer side of the inner membrane of mitochondria. AIFM2 oxidizes NADH to NAD, which is needed for glyceraldehyde-3 phosphate dehydrogenase (GAPDH) reaction, in supporting robust glycolysis. In addition, electrons generated from AIFM2 action are transferred to CoQ for ETC activity of mitochondria. AIFM2 KO mice exhibited higher body weight, lower oxygen consumption, and lower BAT temperature whereas AIFM2 overexpression resulted in the opposite phenotypes. In addition, the expression of AIFM2 was induced upon refeeding with high carb diet when examined at thermoneutrality, suggesting that glucose oxidation appears to be especially important in the fed state to promote thermogenesis when the blood glucose level is relatively high.

Collectively, there is no doubt that β_3_-AR signaling plays a dominant role in regulating thermogenesis. Besides those mentioned above, there are many more proteins that are involved in and downstream of β_3_-AR signaling. Further studies will elucidate more components related to β_3_-AR signaling that regulate thermogenesis at various steps such as substrate metabolism, mitochondrial homeostasis, gene expression as well as signal transduction.

## Insulin/IGF1 Signaling

Insulin/IGF1 signaling plays important roles in adipose tissue development and thermogenesis. Mice with fat-specific double knockout of insulin receptor (IR) and IGF1 receptor (IGF1R) (FIGIRKO) exhibited decreased adiposity with more than 85% reduction in BAT, having lower body weight (35). Indeed, brown preadipocytes from FIGIRKO failed to differentiate *in vitro* with no increase in the expression of PPARγ and CEBPα, indicating that insulin/IGF-1 signaling is critical for BAT development. Since the addition of PPARγ agonist, rosiglitazone, did not rescue the impaired differentiation, insulin/IGF-1 signaling seemed to act on the upstream of PPARγ/CEBPα. Consistently, phosphorylation of CEBPβ at Thr188, which is required for DNA binding and therefore transcriptional activation of PPARγ/CEBPα, was reduced. FIGIRKO mice were also protected from diet-induced obesity, possibly due to impaired adipose development. FIGIRKO mice failed to maintain their body temperature during cold exposure, although they had higher energy expenditure/basal metabolic rate with no change in activity, this paradox remaining to be explained. In this regard, the study utilized aP2 promoter to express Cre recombinase for gene KO, which could have affected not only adipose tissues but also other cell types, such as macrophages.

The effects of insulin signaling and IGF-1 signaling were further investigated by comparing fat-specific IR single KO (F-IRKO) and IGF1R single KO (F-IGFRKO) to IR/IGF1R double KO (F-IR/IGFRKO), which were all generated by Cre driven by adiponectin promoter (36). F-IGFRKO only had a small reduction in both WAT and BAT, while F-IR/IGFRKO had an almost complete loss of adipose tissue. Interestingly, F-IRKO had a large WAT reduction with an unexplainable 50% increase in BAT with large unilocular lipid droplet. BAT of F-IR/IGFRKO exhibited increased BAT gene expression and decreased WAT gene expression, whereas that of F-IRKO showed no change in BAT or WAT marker gene expression. Regardless, the expression of genes involved in BAT function, including UCP1 and other thermogenic genes, TFAM for example, was reduced in both F-IR/IGFRKO and F-IRKO, which could explain why both F-IR/IGFRKO and F-IRKO failed to maintain body temperature during the cold challenge. Indeed, F-IRKO had reduced mitochondrial content and oxygen consumption.

BAT-specific IR KO mice created by using UCP1 promoter-Cre, however, showed impaired BAT development (37). Since the expression of CEBPα, but not PPARγ, decreased in BAT of the KO mice, insulin signaling likely promotes brown adipogenesis mainly through CEBPα. Interestingly, UCP1 expression increased significantly in BAT and the KO mice even had leaner phenotype, possibly due to the remaining BAT compensating for the decreased thermogenesis. Another mechanism where insulin/IGF1 signaling can promote BAT development is by increasing proliferation. Insulin and IGF1, not only stimulated IRS1 and IRS2 tyrosine phosphorylation and their association with PI3K, but also enhanced IRS1-associated GRB2 phosphorylation, which can activate RAS-MAPK pathway for proliferation (38). BAT-specific IGF1R KO mice, on the other hand, had normal BAT size and normal body weight, while UCP1 expression in BAT was reduced and the KO mice were cold intolerant, suggesting that IGF1 signaling enhances BAT thermogenesis (39).

Insulin/IGF1 signaling plays a role in browning of WAT as well. PTEN antagonizes the action of PI3K by converting PIP3 back to PIP2 and, therefore, insulin/IGF1 signaling. PTEN knockdown (KD) by direct injection of Rec2-Cre in iWAT of PTEN floxed mice increased AKT signaling and induced browning (40). These effects were blocked by administration of an AKT inhibitor, indicating that the PI3K-AKT pathway promotes browning. Transgenic mice overexpressing PTEN were reported to have increased energy expenditure with hyperactivation of BAT. However, PTEN was overexpressed globally in these animals, making it difficult to interpret the exact contribution of PTEN in a tissue-specific manner (41).

Interestingly, insulin signaling can be modulated by β_3_-adrenergic signaling. β_3_-adrenergic signaling pretreatment reduced the activation of IR, IRS1 and IRS2. In addition, β_3_-adrenergic signaling mitigated AKT activation and IRS1-associated activation of PI3K upon insulin treatment, while it did not affect MAPK activation and IRS2-associated activation of PI3K. Moreover, β_3_-adrenergic signaling abolished insulin-mediated glucose uptake in brown adipocytes (42). In this regard, cold exposure has also been shown to alter the expression of many molecules involved in the insulin signaling pathway (43). Specifically, the expression of IRS1 and IRS2 as well as phosphorylation of AKT are upregulated. These discrepancies could be due to the differences in the methods used such as *in vitro* vs *in vivo*, mouse vs rat as well as protein phosphorylation vs transcriptome.

Taken together, IR/IGF1R signaling promotes adipose tissue thermogenesis by supporting BAT development, browning of WAT and BAT function. Some of those studies mentioned above are even conflicting, owing at least partly to the differences in promoters used to express Cre, which affects not only the efficiency of gene KO, but also tissue specificity. Even with a tissue-specific KO, some phenotypes could manifest because of secondary effects rather than direct, primary effects. Therefore, it would be important to recapitulate the results *in vitro* as well. Further research is needed to better clarify the role of IR and IGF1R signaling in adipose tissue thermogenesis.

## Thyroid Hormone

Among various ligands/hormones that bind and function *via* nuclear hormone receptors, thyroid hormone (TH) is classically known to regulate thermogenesis (44). TH receptors (TR), TRα and TRβ, in adipocytes enhance BAT thermogenesis and WAT browning. TRs are activated by triiodothyronine (T3), an active form of TH produced from thyroxine (T4) by type II iodothyronine deiodinase (DIO2). Although TRα and TRβ may have overlapping functions, TRβ seems mainly responsible for UCP1 induction, whereas TRα might be more important for full β_3_-adrenergic response. In hypothyroid mice, TRβ-selective ligand, GC-1, restored UCP1 expression to the same extent as T3, suggesting the significant role of TRβ in UCP1 upregulation (45). However, GC-1-treated mice failed to maintain body temperature during cold exposure, suggesting that UCP1 induction by TRβ alone is not sufficient to support thermogenesis. In agreement with this, GC-1 treatment did not restore cAMP production in isolated brown adipocytes of the hypothyroid mice, which suggests the involvement of TRα in β_3_-adrenergic response. The same group later showed that hypothyroid mice with a dominant negative TRβ mutation failed to increase UCP1 expression in BAT (46). These hypothyroid TRβ mutant mice also had reduced cAMP production in BAT and lower body temperature upon T3 replacement and β_3_-adrenergic stimulation, implying that TRβ might at least partly participate in β_3_-adrenergic response. Although it has also been reported that TRα could bind UCP1 enhancer region for transcriptional activation, the results of the study were relied on *in vitro* experiments as opposed to the previously mentioned studies that investigated its role in animal models (47). DIO2 is another important TH/TR target that contributes to thermogenesis. GC-1 upregulated DIO2 mRNA and enzyme activity upon β_3_-adrenergic stimulation, while TRα-selective ligand, CO23, increased DIO2 mRNA expression only (48). Indeed, DIO2 KO brown adipocytes exhibited reduced UCP1 expression, oxygen consumption and lipolysis in response to β3-adrenergic stimuli, which was reversed by T3 administration (49).

TH/TR can enhance BAT thermogenesis through regulation of mitochondrial homeostasis as well (50). Hyperthyroid mice showed increased expression of mitochondrial proteins including COX4I1 and TOMM20 and increased mitochondrial DNA copy number in BAT, implying that TH promoted mitochondrial biogenesis. On the other hand, T3-treated brown adipocytes exhibited mitochondrial remnants inside autophagic vesicles, suggesting that T3 induced mitophagy. Inhibition of autophagy/mitophagy in hyperthyroid mice indeed increased mitochondrial proteins. Mitochondrial turnover was further assessed using MitoTimer, which showed upregulation of both clearance of mature mitochondria and generation of new mitochondria by T3 treatment. The importance of mitochondrial turnover for thermogenesis was confirmed by suppression of autophagy/mitophagy by ATG5 KD, which decreased oxygen consumption in brown adipocytes, and also conditional ATG5 KO in hyperthyroid mice that showed reduced body temperature.

T3 treatment has been shown to upregulate UCP1 and mitochondria-related genes in white adipocytes as well, by increasing mitochondrial biogenesis (51, 52). This was accompanied by elevated oxygen consumption and FA oxidation, indicating the role of T3 in WAT browning. Similar to that observed in brown adipocytes, UCP1 upregulation in WAT was mediated by TRβ rather than TRα since hyperthyroid TRβ KO mice failed to increase UCP1 expression in WAT (53). In addition to directly upregulating UCP1 transcription, T3 was reported also to trigger AMPK activation, which in turn activated the p38/ATF2 pathway to promote UCP1 expression in WAT, although the mechanism by which T3 could activate AMPK remains to be addressed (54).

Recently, a TH metabolite, 3,5 Diiodo-L-Thyronine (T2), was reported to affect thermogenesis as well (55). T2 administration restored lipid morphology, vascularization, innervation and mitochondrial content/activity in BAT of hypothyroid rats, which were accompanied by increased expression of UCP1 and PGC1α, leading to improved thermogenesis (56). Similarly, T2 seems to promote WAT browning in high fat diet-induced obese rats partly through upregulation of PRDM16 (57). Overall, it can be concluded that the TH/TR promotes BAT thermogenesis and WAT browning.

## Transforming Growth Factor β Superfamily

**Figure 2:**
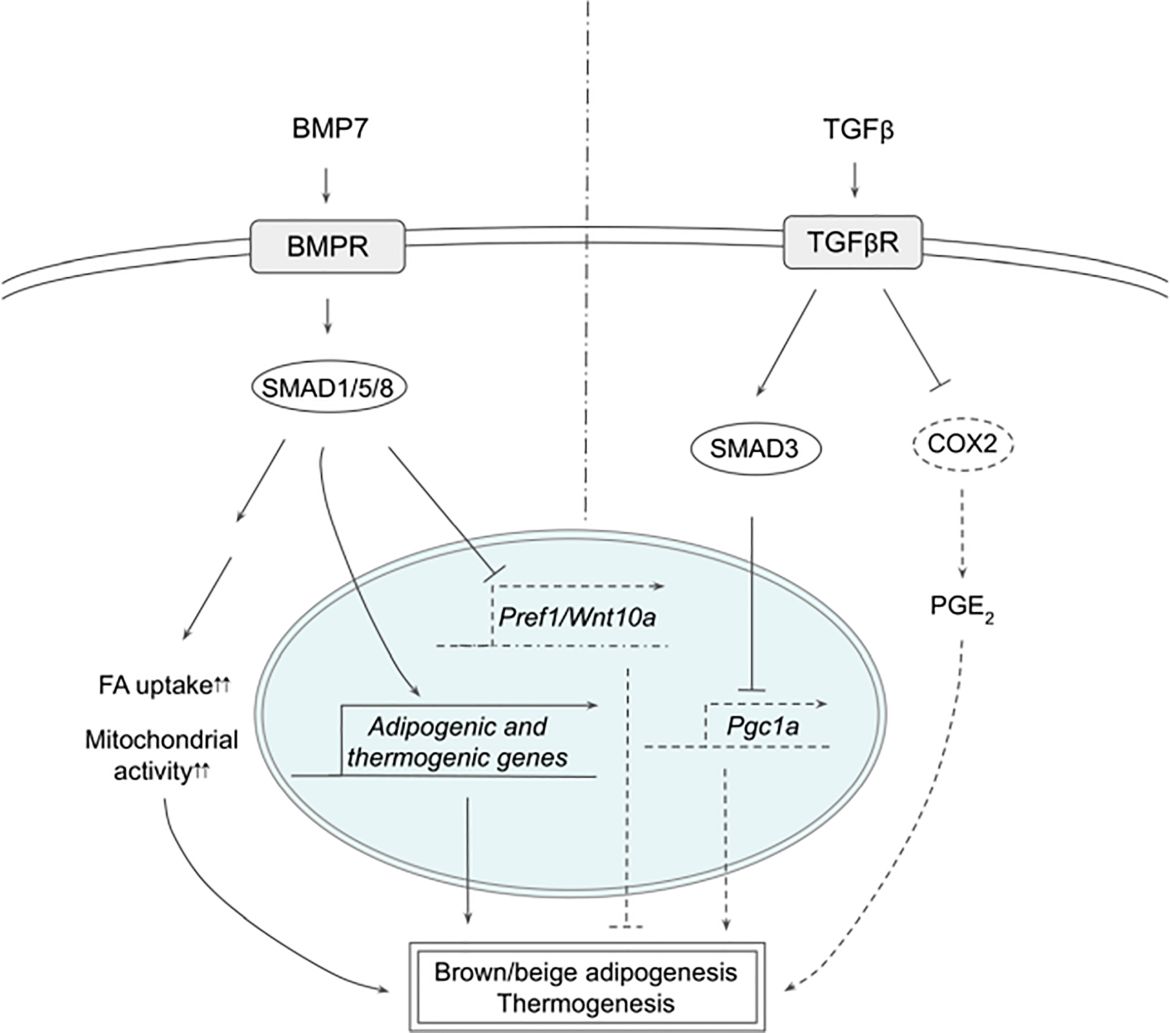
Schematic models of BMP7 and TGFβ signaling pathways in thermogenesis. BMP7 binds TGFβR, which activates SMAD1/5/8, leading to expression of adipogenic and thermogenic genes as well as suppression of Pref1 and Wnt10a in precursor cells to promote brown and beige adipogenesis. In mature adipocytes, BMP7 signaling increases FA uptake and mitochondrial activity, resulting in enhanced thermogenesis. TGFβ activates SMAD2/3 that suppresses PGC1α expression and COX2/PGE2 pathway to reduce thermogenesis. See text for details. BMP7, bone morphogenetic protein 7; COX2, cyclooxygenase 2; FA, fatty acid; PGC1α, peroxisome proliferator activated receptor γ; PGE2, prostaglandin E2; PREF1, preadipocyte factor 1; SMAD, mothers against decapentaplegic homolog; TβR1, TGFβ receptor 1; TGFβ, Transforming growth factor beta; TGFβR, TGFβ receptor; WNT10a, Wnt family member 10A.

Multiple members of the transforming growth factor beta (TGFβ) superfamily have been shown to regulate thermogenesis (**Figure 2**). In fact, bone morphogenetic protein 7 (BMP7), was reported first to trigger commitment of mesenchymal progenitor cells to brown adipocyte lineage by activating SMAD1/5/8 (58). Without the canonical differentiation induction cocktail, BMP7 induced the expression of both adipogenic and brown-adipocyte specific genes, such as CEBPs, PPARγ, PRDM16, PGC1α, and UCP1, while downregulating adipogenic suppressors such as Pref-1 and WNT10A. BMP7 KO mice exhibited reduced BAT mass that had almost undetectable UCP1 expression. In contrast, BMP7 overexpression *via* adenovirus injection increased the expression of PRDM16 and UCP1 in BAT, energy expenditure and body temperature, while decreasing body weight. In mature brown adipocytes, BMP7 increased mitochondrial activity through p38-ATF2 and SMAD1/5/8 pathways (59). BMP7-treated cells upregulated the expression of CD36 and CPT1 that transport FAs into the cells and mitochondria, respectively, resulting in increased citrate synthase activity and FA oxidation. Administration of BMP7 *in vivo* increased the expression of CD36 in BAT, which was accompanied by increased oxygen consumption. In addition, respiratory exchange ratio in these animals decreased, reflecting the substrate utilization switching towards fatty acids. Another study also confirmed the similar effects of BMP7, where BMP7 administration increased the expression of UCP1 and CD36 in BAT, BAT mass, energy expenditure and fat oxidation (60). Furthermore, these authors found that BMP7 induced the expression of BAT genes in WAT depots with overall reduced WAT size, suggesting that BMP7 promotes browning of WAT. This is supported by a finding that demonstrated BMP7 driving human adipogenic stem cells into beige adipocytes (61). BMP7 treatment promoted the expression of beige markers, such as CD137 and TMEM26, in addition to the expression of thermogenic genes, such as UCP1, CIDEA and DIO2.

BMP8b was found to be highly expressed in BAT and its expression increased during differentiation and during cold exposure (62). BMP8b KO mice exhibited lower energy expenditure, lower body temperature and higher body weight, which were exacerbated when these mice were on high fat diet (HFD). Although BMP8b KO mice showed normal BAT size and BAT gene expression, activation of thermogenic signaling pathways examined by phosphorylation of SMAD1/5/8/, p38 and CREB were impaired, indicating that BMP8b is indispensable for thermogenic activation of BAT, but not for BAT development. Consistently, BMP8b treatment increased phosphorylation of these proteins in differentiated brown adipocytes, as well as that of HSL and AMPK, resulting in increased lipolysis measured by glycerol release. In addition, BMP8b promoted the activation of AMPK in the central nervous system as well, increasing sympathetic output to BAT to support thermogenesis. In this regard, BMP8b was shown to stimulate sympathetic innervation and vascularization through NRG4 and VEGF, respectively, in both BAT and WAT as well as browning of WAT (63). Mice overexpressing BMP8b showed higher energy expenditure, lower body weight and fat mass, accompanied by decreased thermogenic gene expression in WAT that exhibited multilocular lipid droplets, whereas loss of BMP8b resulted in decreased nerve density.

TGFβ/SMAD3 signaling pathway has been reported to suppress browning of WAT (64). TGFβ treatment suppressed activation of PGC1α promoter-reporter in 3T3-L1 cells, which was abolished by SMAD3 KD, indicating that TGFβ represses PGC1α transcription through SMAD3. SMAD3 KO animals had higher body temperature, lower body weight, lower blood glucose, and better response to insulin. WAT of SMAD3 KO mice indeed expressed thermogenic genes, such as UCP1, and exhibited multilocular morphology. Similarly, adipose-specific TGFβ receptor 1 (TGFβR1) KO induced browning, as evidenced by increased UCP1 expression in WAT on HFD, which was accompanied by lower body weight (65). TGFβR1 inhibitor, SB431542, induced brown and beige genes during differentiation of WAT SVF cells, whereas TβR1 downregulated those genes. Interestingly, WAT of TGFβR1 KO also increased the expression of genes involved in prostaglandin pathways, such as COX2, implying that prostaglandin pathway might be involved in browning of WAT. SMAD3 KD in 3T3-L1 cells increased the expression of COX2 and other prostaglandin pathway-related proteins. Treatment with PGE_2_, a product of COX2, upregulated thermogenic gene expression, such as CIDEA1 and PGC1α, in the presumptive beige precursor cells, which was further augmented by the addition of SB431542, implying the possible interaction between TGFβ/SMAD3 and COX2/PGE_2_ pathways. These presumptive beige precursor cells were isolated and selected from WAT by FACS using a series of cell surface markers and were termed inducible beige stem/progenitor cells (iBSCs). iBSCs were then transplanted subcutaneously into immunodeficient nude mice on HFD with IPTT-300 transponder. During the cold exposure, iBSCs from TβR1 KO mice maintained higher temperature and showed higher expression of thermogenic genes with multilocular lipid droplet morphology, suggesting that the KO iBSCs underwent beige adipogenesis. Altogether, TGFβ/SMAD3 signaling negatively regulates WAT browning although further research will help to clarify the contributions of different downstream pathways.

## Concluding Remark

The discovery of brown or brown-like adipocytes in human adults has made targeting adipocytes an attractive strategy to fight against obesity and its associated diseases. Activation of existing thermogenic adipocytes and/or induction of new thermogenic adipocytes can be mediated by various hormones and their signaling cascades, including those important pathways we describe in this review. Understanding the precise molecular mechanisms underlying regulation of thermogenesis and browning of WAT is critical for advancement of the future anti-obesity therapeutics.

## Author Contributions

CT drafted and HS revised the manuscript. All authors contributed to the article and approved the submitted version.

## Funding

The work was supported in part by DK120075 to HSS.

## Conflict of Interest

The authors declare that the research was conducted in the absence of any commercial or financial relationships that could be construed as a potential conflict of interest.

## References

[1] Nedergaard J Cannon B. Brown adipose tissue as a heat-producing thermoeffector. Handb Clin Neurol (2018) 156:137–52. 10.1016/B978-0-444-63912-7.00009-6 30454587

[2] Lidell ME. Brown Adipose Tissue in Human Infants. In: Pfeifer A Klingenspor M Herzig S, editors. Brown Adipose Tissue. Cham: Springer International Publishing (2019). p. 107–23. 10.1007/164_2018_118 29675580

[3] Wang Q Zhang M Xu M Gu W Xi Y Qi L. Brown adipose tissue activation is inversely related to central obesity and metabolic parameters in adult human. PloS One (2015) 10(4):e0123795. 10.1371/journal.pone.0123795 25894250 PMC4403996

[4] Saito M. Brown adipose tissue as a regulator of energy expenditure and body fat in humans. Diabetes Metab J (2013) 37(1):22–9. 10.4093/dmj.2013.37.1.22 PMC3579148 23441053

[5] Fenzl A Kiefer FW. Brown adipose tissue and thermogenesis. Hormone Mol Biol Clin Invest (2014) 19(1):1868–91. 10.1515/hmbci-2014-0022 25390014

[6] Lattin JE Schroder K Su AI Walker JR Zhang J Wiltshire T. Expression analysis of G Protein-Coupled Receptors in mouse macrophages. Immunome Res (2008) 4:5. 10.1186/1745-7580-4-5 18442421 PMC2394514

[7] Chouchani ET Kazak L Spiegelman BM. New Advances in Adaptive Thermogenesis: UCP1 and Beyond. Cell Metab (2018) 29(1):27–37. 10.1016/j.cmet.2018.11.002 30503034

[8] Herz CT Kiefer FW. Adipose tissue browning in mice and humans. J Endocrinol (2019) 241(3):R97–109. 10.1530/JOE-18-0598 31144796

[9] Wu J Boström P Sparks LM Ye L Choi JH Giang A-H. Beige adipocytes are a distinct type of thermogenic fat cell in mouse and human. Cell (2012) 150(2):366–76. 10.1016/j.cell.2012.05.016 PMC3402601 22796012

[10] Shao M Wang QA Song A Vishvanath L Busbuso NC Scherer PE. Cellular Origins of Beige Fat Cells Revisited. Diabetes (2019) 68(10):1874–85. 10.2337/db19-0308 PMC6754244 31540940

[11] Wang W Seale P. Control of brown and beige fat development. Nat Rev Mol Cell Biol (2016) 17(11):691–702. 10.1038/nrm.2016.96 27552974 PMC5627770

[12] Rajakumari S Wu J Ishibashi J Lim H-W Giang A-H Won K-J. EBF2 determines and maintains brown adipocyte identity. Cell Metab (2013) 17(4):562–74. 10.1016/j.cmet.2013.01.015 PMC3622114 23499423

[13] Seale P Bjork B Yang W Kajimura S Chin S Kuang S. PRDM16 controls a brown fat/skeletal muscle switch. Nature (2008) 454(7207):961–7. 10.1038/nature07182 PMC2583329 18719582

[14] Park JH Kang HJ Kang SI Lee JE Hur J Ge K. A multifunctional protein, EWS, is essential for early brown fat lineage determination. Dev Cell (2013) 26(4):393–404. 10.1016/j.devcel.2013.07.002 23987512 PMC3817832

[15] Inagaki T Sakai J Kajimura S. Transcriptional and epigenetic control of brown and beige adipose cell fate and function. Nat Rev Mol Cell Biol (2016) 17(8):480–95. 10.1038/nrm.2016.62 PMC4956538 27251423

[16] Dempersmier J Sambeat A Gulyaeva O Paul SM Hudak CSS Raposo HF. Cold-inducible Zfp516 activates UCP1 transcription to promote browning of white fat and development of brown fat. Mol Cell (2015) 57(2):235–46. 10.1016/j.molcel.2014.12.005 PMC4304950 25578880

[17] Yi D Dempersmier JM Nguyen HP Viscarra JA Dinh J Tabuchi C. Zc3h10 Acts as a Transcription Factor and Is Phosphorylated to Activate the Thermogenic Program. Cell Rep (2019) 29(9):2621–33.e4. 10.1016/j.celrep.2019.10.099 31775033 PMC6911170

[18] Koppen A Kalkhoven E. Brown vs white adipocytes: the PPARgamma coregulator story. FEBS Lett (2010) 584(15):3250–9. 10.1016/j.febslet.2010.06.035 20600006

[19] Kajimura S Seale P Kubota K Lunsford E Frangioni JV Gygi SP. Initiation of myoblast to brown fat switch by a PRDM16-C/EBP-beta transcriptional complex. Nature (2009) 460(7259):1154–8. 10.1038/nature08262 PMC2754867 19641492

[20] Gill JA La Merrill MA. An emerging role for epigenetic regulation of Pgc-1α expression in environmentally stimulated brown adipose thermogenesis. Environ Epigenet (2017) 3(2):dvx009. 10.1093/eep/dvx009 29492311 PMC5804549

[21] Ikeda K Maretich P Kajimura S. The Common and Distinct Features of Brown and Beige Adipocytes. Trends Endocrinol Metab (2018) 29(3):191–200. 10.1016/j.tem.2018.01.001 29366777 PMC5826798

[22] Yi D Nguyen HP Sul HS. Epigenetic dynamics of the thermogenic gene program of adipocytes. Biochem J (2020) 477(6):1137–48. 10.1042/BCJ20190599 PMC8594062 32219439

[23] Reverte-Salisa L Sanyal A Pfeifer A. Brown Adipose Tissue: Role of cAMP and cGMP Signaling in Brown Fat. In: Pfeifer A Klingenspor M Herzig S, editors. Brown Adipose Tissue. Cham: Springer International Publishing (2019). p. 161–82. 10.1007/164_2018_117 29633180

[24] Liu J Wang Y Lin L. Small molecules for fat combustion: targeting obesity. Acta Pharm Sin B (2019) 9(2):220–36. 10.1016/j.apsb.2018.09.007 PMC6438825 30976490

[25] Schena G Caplan MJ. Everything You Always Wanted to Know about β3-AR * (* But Were Afraid to Ask). Cells (2019) 8(4):357. 10.3390/cells8040357 PMC6523418 30995798

[26] Shi F Collins S. Second messenger signaling mechanisms of the brown adipocyte thermogenic program: an integrative perspective. Hormone Mol Biol Clin Invest (2017) 31(2):1868–91. 10.1515/hmbci-2017-0062 28949928

[27] Cao W Daniel KW Robidoux J Puigserver P Medvedev AV Bai X. p38 mitogen-activated protein kinase is the central regulator of cyclic AMP-dependent transcription of the brown fat uncoupling protein 1 gene. Mol Cell Biol (2004) 24(7):3057–67. 10.1128/MCB.24.7.3057-3067.2004 PMC371122 15024092

[28] Liang H Ward WF. PGC-1alpha: a key regulator of energy metabolism. Adv Physiol Educ (2006) 30(4):145–51. 10.1152/advan.00052.2006 17108241

[29] Symonds ME. Adipose Tissue Biology. Cham: Springer (2017).

[30] Kajimura S Saito M. A new era in brown adipose tissue biology: molecular control of brown fat development and energy homeostasis. Annu Rev Physiol (2014) 76:225–49. 10.1146/annurev-physiol-021113-170252 PMC4090362 24188710

[31] Blondin DP Frisch F Phoenix S Guérin B Turcotte ÉE Haman F. Inhibition of Intracellular Triglyceride Lipolysis Suppresses Cold-Induced Brown Adipose Tissue Metabolism and Increases Shivering in Humans. Cell Metab (2017) 25(2):438–47. 10.1016/j.cmet.2016.12.005 28089568

[32] Shin H Ma Y Chanturiya T Cao Q Wang Y Kadegowda AKG. Lipolysis in Brown Adipocytes Is Not Essential for Cold-Induced Thermogenesis in Mice. Cell Metab (2017) 26(5):764–77.e5. 10.1016/j.cmet.2017.09.002 28988822 PMC5905336

[33] Braun K Oeckl J Westermeier J Li Y Klingenspor M. Non-adrenergic control of lipolysis and thermogenesis in adipose tissues. J Exp Biol (2018) 221(Pt Suppl 1):jeb165381. 10.1242/jeb.165381 29514884

[34] Nguyen HP Yi D Lin F Viscarra JA Tabuchi C Ngo K. Aifm2, a NADH Oxidase, Supports Robust Glycolysis and Is Required for Cold- and Diet-Induced Thermogenesis. Mol Cell (2020) 77(3):600–17.e4. 10.1016/j.molcel.2019.12.002 31952989 PMC7031813

[35] Boucher J Mori MA Lee KY Smyth G Liew CW Macotela Y. Impaired thermogenesis and adipose tissue development in mice with fat-specific disruption of insulin and IGF-1 signalling. Nat Commun (2012) 3:902. 10.1038/ncomms1905 22692545 PMC3529640

[36] Boucher J Softic S El Ouaamari A Krumpoch MT Kleinridders A Kulkarni RN. Differential Roles of Insulin and IGF-1 Receptors in Adipose Tissue Development and Function. Diabetes (2016) 65(8):2201–13. 10.2337/db16-0212 PMC4955980 27207537

[37] Guerra C Navarro P Valverde AM Arribas M Brüning J Kozak LP. Brown adipose tissue-specific insulin receptor knockout shows diabetic phenotype without insulin resistance. J Clin Invest (2001) 108(8):1205–13. 10.1172/JCI13103 PMC209529 11602628

[38] Valverde AM Lorenzo M Pons S White MF Benito M. Insulin receptor substrate (IRS) proteins IRS-1 and IRS-2 differential signaling in the insulin/insulin-like growth factor-I pathways in fetal brown adipocytes. Mol Endocrinol (1998) 12(5):688–97. 10.1210/mend.12.5.0106 9605931

[39] Viana-Huete V Guillén C García-Aguilar A García G Fernández S Kahn CR. Essential Role of IGFIR in the Onset of Male Brown Fat Thermogenic Function: Regulation of Glucose Homeostasis by Differential Organ-Specific Insulin Sensitivity. Endocrinology (2016) 157(4):1495–511. 10.1210/en.2015-1623 PMC6285213 26910308

[40] Huang W Queen NJ McMurphy TB Ali S Cao L. Adipose PTEN regulates adult adipose tissue homeostasis and redistribution via a PTEN-leptin-sympathetic loop. Mol Metab (2019) 30:48–60. 10.1016/j.molmet.2019.09.008 31767180 PMC6812328

[41] Ortega-Molina A Efeyan A Lopez-Guadamillas E Muñoz-Martin M Gómez-López G Cañamero M. Pten positively regulates brown adipose function, energy expenditure, and longevity. Cell Metab (2012) 15(3):382–94. 10.1016/j.cmet.2012.02.001 22405073

[42] Klein J Fasshauer M Ito M Lowell BB Benito M Kahn CR. β3-Adrenergic Stimulation Differentially Inhibits Insulin Signaling and Decreases Insulin-induced Glucose Uptake in Brown Adipocytes. J Biol Chem (1999) 274(49):34795–802. 10.1074/jbc.274.49.34795 10574950

[43] Wang X Wahl R. Responses of the insulin signaling pathways in the brown adipose tissue of rats following cold exposure. PloS One (2014) 9(6):e99772. 10.1371/journal.pone.0099772 24915042 PMC4051765

[44] Yau WW Yen PM. Thermogenesis in Adipose Tissue Activated by Thyroid Hormone. Int J Mol Sci (2020) 21(8):3020. 10.3390/ijms21083020 PMC7215895 32344721

[45] Ribeiro MO Carvalho SD Schultz JJ Chiellini G Scanlan TS Bianco AC. Thyroid hormone–sympathetic interaction and adaptive thermogenesis are thyroid hormone receptor isoform–specific. J Clin Invest (2001) 108(1):97–105. 10.1172/JCI200112584 11435461 PMC209342

[46] Ribeiro MO Bianco SDC Kaneshige M Schultz JJ Cheng S-Y Bianco AC. Expression of uncoupling protein 1 in mouse brown adipose tissue is thyroid hormone receptor-beta isoform specific and required for adaptive thermogenesis. Endocrinology (2010) 151(1):432–40. 10.1210/en.2009-0667 PMC2817565 19906816

[47] Chen W Yang Q Roeder RG. Dynamic interactions and cooperative functions of PGC-1alpha and MED1 in TRalpha-mediated activation of the brown-fat-specific UCP-1 gene. Mol Cell (2009) 35(6):755–68. 10.1016/j.molcel.2009.09.015 PMC3217464 19782026

[48] Martinez de Mena R Scanlan TS Obregon M-J. The T3 receptor beta1 isoform regulates UCP1 and D2 deiodinase in rat brown adipocytes. Endocrinology (2010) 151(10):5074–83. 10.1210/en.2010-0533 20719854

[49] de Jesus LA Carvalho SD Ribeiro MO Schneider M Kim SW Harney JW. The type 2 iodothyronine deiodinase is essential for adaptive thermogenesis in brown adipose tissue. J Clin Invest (2001) 108(9):1379–85. 10.1172/JCI200113803 PMC209445 11696583

[50] Yau WW Singh BK Lesmana R Zhou J Sinha RA Wong KA. Thyroid hormone (T3) stimulates brown adipose tissue activation via mitochondrial biogenesis and MTOR-mediated mitophagy. Autophagy (2019) 15(1):131–50. 10.1080/15548627.2018.1511263 PMC6287687 30209975

[51] Lee J-Y Takahashi N Yasubuchi M Kim Y-I Hashizaki H Kim M-J. Triiodothyronine induces UCP-1 expression and mitochondrial biogenesis in human adipocytes. Am J Physiol Cell Physiol (2012) 302(2):C463–72. 10.1152/ajpcell.00010.2011 22075692

[52] Krause K. Novel Aspects of White Adipose Tissue Browning by Thyroid Hormones. Exp Clin Endocrinol Diabetes (2020) 128(6-07):446–9. 10.1055/a-1020-5354 31698480

[53] Johann K Cremer AL Fischer AW Heine M Pensado ER Resch J. Thyroid-Hormone-Induced Browning of White Adipose Tissue Does Not Contribute to Thermogenesis and Glucose Consumption. Cell Rep (2019) 27(11):3385–400.e3. 10.1016/j.celrep.2019.05.054 31189119

[54] Matesanz N Bernardo E Acín-Pérez R Manieri E Pérez-Sieira S Hernández-Cosido L. MKK6 controls T3-mediated browning of white adipose tissue. Nat Commun (2017) 8(1):856. 10.1038/s41467-017-00948-z 29021624 PMC5636784

[55] Louzada RA Carvalho DP. Similarities and Differences in the Peripheral Actions of Thyroid Hormones and Their Metabolites. Front Endocrinol (2018) 9:394. 10.3389/fendo.2018.00394 PMC6060242 30072951

[56] Lombardi A Senese R De Matteis R Busiello RA Cioffi F Goglia F. 3,5-Diiodo-L-thyronine activates brown adipose tissue thermogenesis in hypothyroid rats. PloS One (2015) 10(2):e0116498. 10.1371/journal.pone.0116498 25658324 PMC4319745

[57] Senese R Cioffi F De Matteis R Petito G de Lange P Silvestri E. 3,5 Diiodo-l-Thyronine (T₂) Promotes the Browning of White Adipose Tissue in High-Fat Diet-Induced Overweight Male Rats Housed at Thermoneutrality. Cells (2019) 8(3):256. 10.3390/cells8030256 PMC6468521 30889829

[58] Tseng Y-H Kokkotou E Schulz TJ Huang TL Winnay JN Taniguchi CM. New role of bone morphogenetic protein 7 in brown adipogenesis and energy expenditure. Nature (2008) 454(7207):1000–4. 10.1038/nature07221 PMC2745972 18719589

[59] Townsend KL An D Lynes MD Huang TL Zhang H Goodyear LJ. Increased mitochondrial activity in BMP7-treated brown adipocytes, due to increased CPT1- and CD36-mediated fatty acid uptake. Antioxid Redox Signal (2013) 19(3):243–57. 10.1089/ars.2012.4536 PMC3691916 22938691

[60] Boon MR van den Berg SAA Wang Y van den Bossche J Karkampouna S Bauwens M. BMP7 activates brown adipose tissue and reduces diet-induced obesity only at subthermoneutrality. PloS One (2013) 8(9):e74083. 10.1371/journal.pone.0074083 24066098 PMC3774620

[61] Okla M Ha J-H Temel RE Chung S. BMP7 drives human adipogenic stem cells into metabolically active beige adipocytes. Lipids (2015) 50(2):111–20. 10.1007/s11745-014-3981-9 PMC4306630 25534037

[62] Whittle AJ Carobbio S Martins L Slawik M Hondares E Vázquez MJ. BMP8B increases brown adipose tissue thermogenesis through both central and peripheral actions. Cell (2012) 149(4):871–85. 10.1016/j.cell.2012.02.066 PMC3383997 22579288

[63] Pellegrinelli V Peirce VJ Howard L Virtue S Türei D Senzacqua M. Adipocyte-secreted BMP8b mediates adrenergic-induced remodeling of the neuro-vascular network in adipose tissue. Nat Commun (2018) 9(1):4974. 10.1038/s41467-018-07453-x 30478315 PMC6255810

[64] Yadav H Quijano C Kamaraju AK Gavrilova O Malek R Chen W. Protection from obesity and diabetes by blockade of TGF-β/Smad3 signaling. Cell Metab (2011) 14(1):67–79. 10.1016/j.cmet.2011.04.013 21723505 PMC3169298

[65] Wankhade UD Lee J-H Dagur PK Yadav H Shen M Chen W. TGF-β receptor 1 regulates progenitors that promote browning of white fat. Mol Metab (2018) 16:160–71. 10.1016/j.molmet.2018.07.008 PMC6158128 30100246

