# Corrigendum: Signaling Pathways Regulating Thermogenesis

**DOI:** 10.3389/fendo.2021.698619

**Published:** 2021-06-22

**Authors:** Chihiro Tabuchi, Hei Sook Sul

**Affiliations:** Department of Nutritional Sciences and Toxicology, University of California, Berkeley, Berkeley, CA, United States

**Keywords:** thermogenesis, brown adipose tissue, browning/beiging, β3-adrenergic signaling, UCP1, insulin/IGF1 signaling, thyroid hormone, TGFβ superfamily

In the original article, there were errors in **Figures 1** and **2**. In **Figure 1**, IRF4 had to be removed and the order of the transcription factors had to be reorganized during this correction**.** In **Figure 2**, SMAD2 needs to be removed. The corrected **Figures 1** and **2** are below.

**Figure 1:**
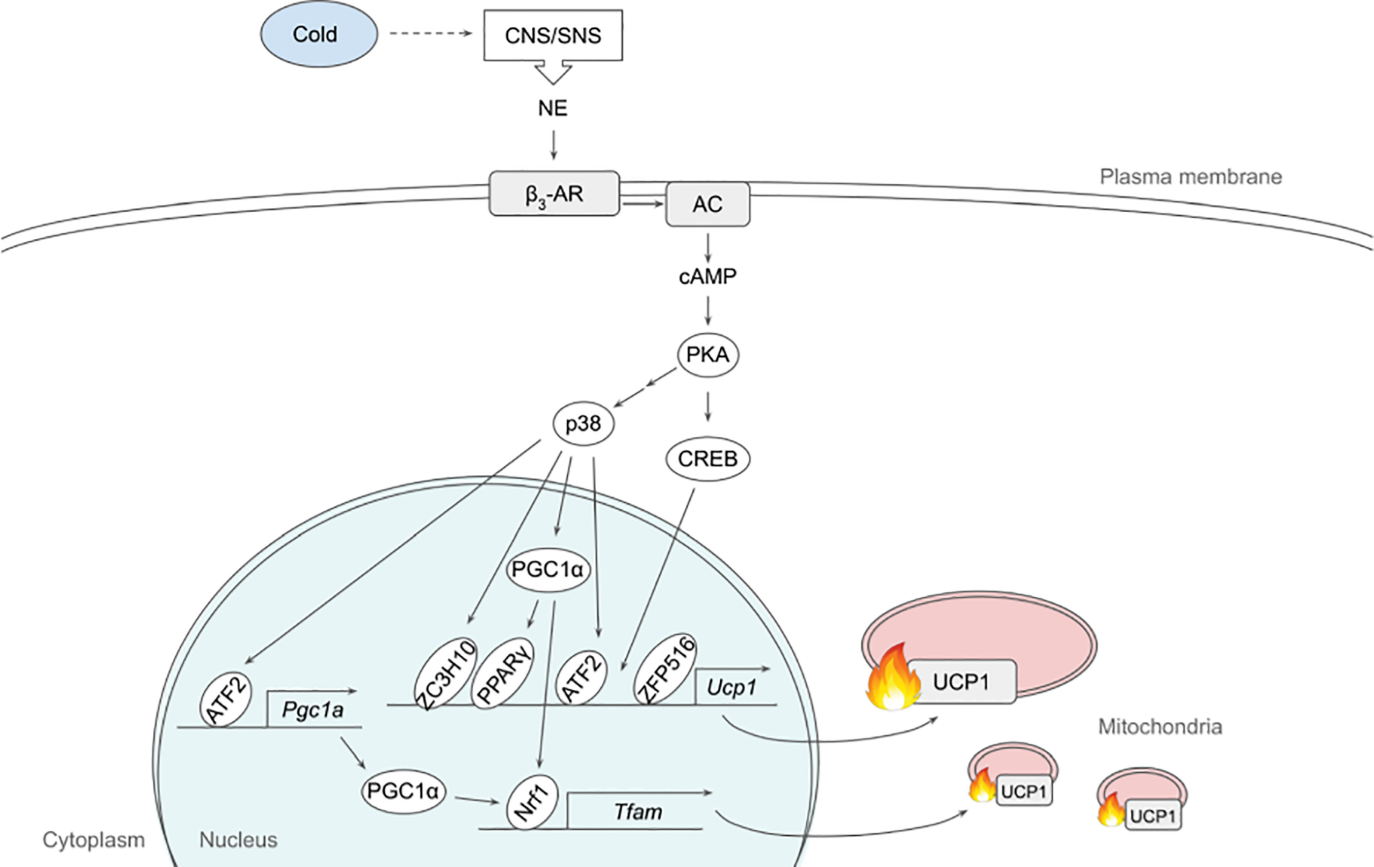
Schematic model of β_3_-adrenergic signaling pathway that promotes thermogenesis in adipocytes. Cold stimulates CNS/SNS to secrete NE that binds β_3_-AR, which then activates AC producing cAMP. cAMP in turn activates PKA that has a variety of downstream targets, including transcription factors to upregulate thermogenic gene expression. See text for details. AC, adenylate cyclase; ATF2, activating transcription factor 2; β_3_-AR, β_3_-adrenergic receptor; CNS, central nervous system; CREB, cAMP response-element binding protein; ETC, electron transport chain; FFA, free fatty acid; IRF4, interferon regulatory factor 4; NE, norepinephrine; NRF1, nuclear respiratory factor 1; PGC1α, peroxisome proliferator activated receptor γ coactivator α; PKA, protein kinase A; PPARγ, peroxisome proliferator activated receptor γ; SNS sympathetic nervous system; TCA, tricarboxylic acid; TFAM, mitochondrial transcription factor A; UCP1, uncoupling protein 1; ZC3H10, zinc finger CCCH-type containing 10; ZFP516, zinc finger protein 516.

**Figure 2:**
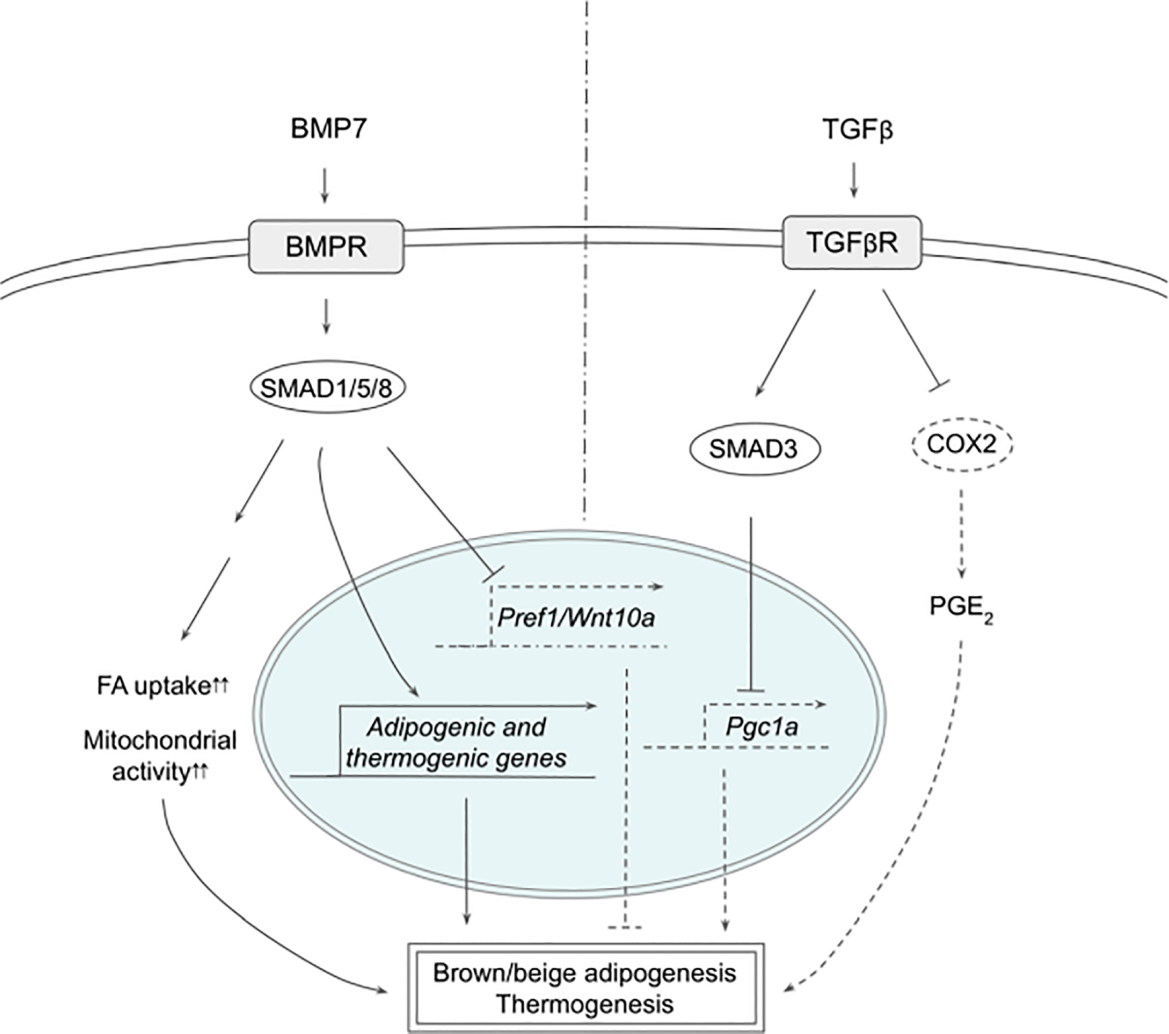
Schematic models of BMP7 and TGFβ signaling pathways in thermogenesis. BMP7 binds TGFβR, which activates SMAD1/5/8, leading to expression of adipogenic and thermogenic genes as well as suppression of Pref1 and Wnt10a in precursor cells to promote brown and beige adipogenesis. In mature adipocytes, BMP7 signaling increases FA uptake and mitochondrial activity, resulting in enhanced thermogenesis. TGFβ activates SMAD2/3 that suppresses PGC1α expression and COX2/PGE2 pathway to reduce thermogenesis. See text for details. BMP7, bone morphogenetic protein 7; COX2, cyclooxygenase 2; FA, fatty acid; PGC1α, peroxisome proliferator activated receptor γ; PGE2, prostaglandin E2; PREF1, preadipocyte factor 1; SMAD, mothers against decapentaplegic homolog; TβR1, TGFβ receptor 1; TGFβ, Transforming growth factor beta; TGFβR, TGFβ receptor; WNT10a, Wnt family member 10A.

In the original article, there were errors in the text. IRF4 needed to be replaced with PGC1α as a downstream target of p38. PGC1α and DIO needed to be removed as downstream targets of CREB, due to insufficient research.

A correction has been made to the section **β_3_-Adrenergic Signaling**, the second paragraph:

“p38, a MAP kinase, phosphorylates multiple transcription factors/coregulators, including ATF2 and PGC1α, both of which promote UCP1 transcription (22). In addition, we recently found that ZC3H10, previously known to bind RNA, is phosphorylated by p38 upon cold, activating the thermogenic gene program in adipocytes (17). Specifically, ZC3H10 binds a distal upstream region of the UCP1 promoter for transcriptional activation. ZC3H10 also activates NRF1 and TFAM, which facilitate mitochondrial biogenesis to increase thermogenic capacity of adipocytes. Thus, transgenic mice overexpressing ZC3H10 exhibited increased oxygen consumption, higher BAT temperature and reduced body weight while ZC3H10 knockout mice displayed decreased oxygen consumption, lower BAT temperature and increased body weight. PGC1α, a downstream target of p38, serves as co-activator for PPARγ and NRF1 to induce expression of UCP1 and TFAM, respectively (27, 28). CREB was also shown to bind to the proximal promoter of UCP1 to potentially activate the transcription.”

The authors apologize for these errors and state that this does not change the scientific conclusions of the article in any way. The original article has been updated.
